# Associations between mechanical stress and epigenetic modifications in outer retinal layer remodeling in high myopia

**DOI:** 10.3389/fmed.2026.1780606

**Published:** 2026-03-31

**Authors:** Jiayu Xu, Liyuan Wang, Zhihua Shen

**Affiliations:** 1 Department of Ophthalmology, Shenzhen Traditional Chinese Medicine Hospital, Shenzhen, China; 2 Heilongjiang University of Chinese Medicine, Harbin, China; 3 Department of Ophthalmology, First Affiliated Hospital of Heilongjiang University of Chinese Medicine, Harbin, China

**Keywords:** epigenetic modifications, FDM, high myopia, mechanical stress, remodeling of outer retinal layers

## Abstract

High myopia is characterized not only by a progressive increase in refractive error but also, more critically, by its significant association with retinal degenerative diseases, posing a considerable public health concern. An increasing amount of research illustrates the role of several epigenetic regulatory mechanisms—including DNA methylation, modifications of histones, non-coding RNAs, and additional epigenetic elements—in the onset and advancement of high myopia. This condition primarily results from the pathological elongation of the eye axis, which alters the mechanical stress on the fundus and accelerates degenerative changes in the retina. The degenerative alterations include modifications in the structure and morphology of retinal pigment epithelial (RPE) cells, along with the restructuring of the outer layers of the retina. Notably, the mechanical stimuli induced by these biomechanical changes can create a form of cellular memory that continues to influence RPE cell behavior even after the initial force is removed. Cells may sense mechanical signals directly through cytoskeleton-associated membrane receptors or indirectly via intracellular biochemical cascades that modulate gene expression in response to environmental cues. This review proposes a conceptual framework to examine the central role of epigenetic modifications in retinal degeneration associated with high myopia. It highlights that RPE cells function as key responders to mechanical stress, capable of forming cellular memory and adaptive responses that drive outer retinal remodeling and exacerbate degenerative processes. This research aims to enhance the comprehension of the pathogenesis related to retinal degenerative alterations linked to high myopia by clarifying the processes involved in mechanical signal transduction and the epigenetic regulation of cellular functions.

## 1 Introduction

Myopia represents a major public health issue worldwide, especially in younger demographics (1). Nonetheless, the exact rise in myopia prevalence remains unclear. Forecasts indicate that by the year 2050, approximately 938 million individuals may be affected by high myopia. Additionally, it is anticipated that the incidence of high myopia in adolescents between 16 and 18 years old will increase from 7.3% in 2001 to 22.1% by 2050 (2). Severe myopia can lead to the deterioration of refractive errors and significantly increase the likelihood of serious ocular complications (3). These include cataracts, glaucoma, retinal detachment, and myopic maculopathy, all of which can result in permanent vision loss (4). Currently, there are no targeted pharmacological interventions available for the early retinal degenerative changes associated with high myopia, which underscores an urgent unmet clinical need.

The majority of individuals experience myopia development influenced by a mix of genetic and environmental elements, along with their interactions (5). Recent studies have shed light on the mechanisms by which these genetic and environmental factors converge to impact the onset and progression of myopia (6). Epigenetics refers to heritable modifications in gene expression that occur due to changes in genomic structure, independent of alterations to the DNA sequence itself (7). Both intrinsic and environmental elements play a role in regulating epigenetic processes, collectively influencing the activation or suppression of particular target genes. Principal molecular mechanisms governing epigenetic regulation in the retina encompass DNA methylation, histone modifications, chromatin remodeling, microRNAs, long non-coding RNAs, and nuclear hormone receptors (8). Furthermore, transcriptomics (RNA methylation) is a field that has received significant attention in recent years regarding gene expression regulation. Common modifications such as m6A and m5C can regulate the stability, splicing, and translation efficiency of mRNA through an “writing-reading-removal” enzyme system, thereby rapidly modulating the post-transcriptional responses of cells (9). Disruption of these epigenetic processes has been associated with a range of retinal disorders, including diabetic retinopathy, retinitis pigmentosa, and age-related macular degeneration (10).

The length of the eye’s axis is crucial in the development of high myopia (11, 12). An ongoing increase in the ocular axis may exert mechanical pressure on the retina, both directly and indirectly (13). High myopia is primarily marked by significant and prolonged enlargement of the eyeball, which is linked to degenerative alterations in the posterior part of the eye (13). In the phase of ongoing posterior elongation, the retina undergoes excessive mechanical stretching, and the microenvironment around retinal cells—encompassing factors like compressive forces, stiffness of the matrix, tensile and shear stresses—is considerably changed, impacting cellular characteristics and facilitating retinal remodeling (14, 15). These mechanical influences may affect various elements of cellular activity, such as cytoskeletal structure, mechanics of the nucleus, mechanosensitive signaling pathways, cellular tension, and modifications in the surrounding extracellular matrix (16). The mechanical transduction caused by mechanics can trigger a cascade of biochemical signals within the cell or cause the deformation of the cell nucleus, thereby regulating the epigenetic state (17). In addition, the chemical and physical signals elicited by mechanical stimulation regulate cellular gene expression patterns through chromatin remodeling enzymes and transcriptional mechanisms (18). Meanwhile, epigenetic regulation can modulate chromatin structure through post-translational modifications of histone proteins, including the methylation and acetylation of specific amino acid residues (19, 20). Histone acetylation and deacetylation regulate the overall chromatin architecture and influence the transcriptionally active regions of genes (21). DNA methylation and demethylation are also implicated in the regulation of chromatin condensation, a process closely associated with cellular metabolism and behavior (22). Overall, these epigenetic modifications may be induced by alterations in the mechanical environment and can influence cellular gene expression, thereby further contributing to the progression of retinal tissue damage.

At present, there is a notable lack of research focused on the intricate interactions between epigenetic mechanisms and mechanical forces in the outer retina’s pathophysiology associated with high myopia and retinal degeneration. This investigation clarifies how epigenetic changes induced by mechanical forces regulate gene expression, cellular activities, and pathological conditions in the outer retina, thus presenting promising directions for further research. This article is a narrative review intended to provide an expert overview rather than an exhaustive systematic synthesis; therefore, no formal systematic search strategy or PRISMA flow diagram was applied. Relevant peer-reviewed literature was identified through targeted searches and citation tracking, prioritizing seminal and recent high-quality studies aligned with the scope of this review.

## 2 Remodeling of the retinal outer layer in high myopia-associated retinal degeneration

**Figure 1:**
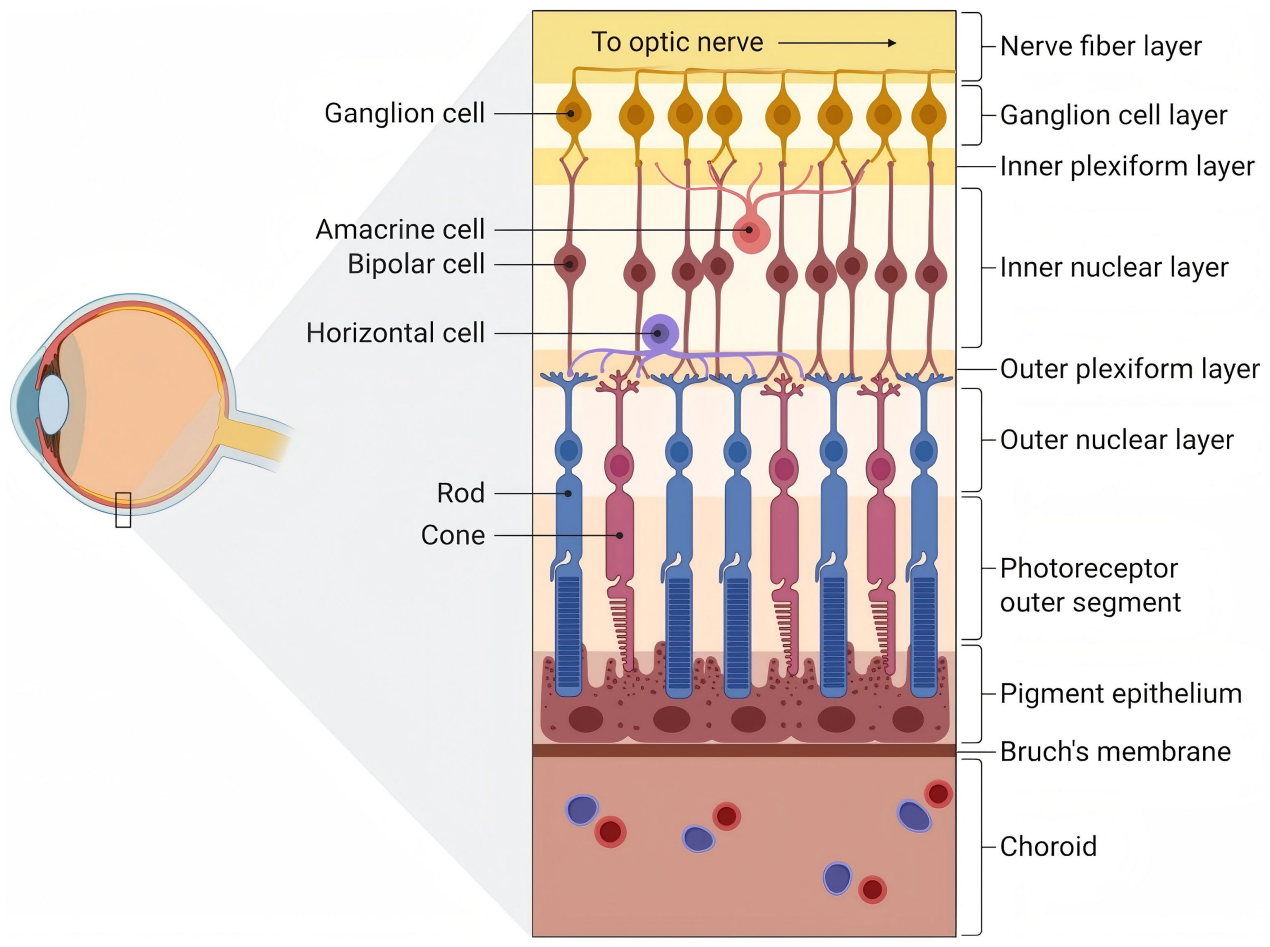
The outer structure of the retina in its physiological state. The photoreceptor outer segment layer consists of the light-sensitive outer segments of rods (blue; responsible for scotopic vision) and cones (pink; responsible for photopic and color vision). The retinal pigment epithelium underlies the photoreceptors and performs essential support functions including phagocytosis of shed outer segments, visual cycle maintenance, and ion transport. Bruch’s membrane separates the retinal pigment epithelium from the choroid, a highly vascularized layer (depicted with red and blue erythrocytes representing oxygenated and deoxygenated blood, respectively) that supplies metabolic support to the outer retina. Created with BioRender.com.

The outer layer of the retina plays a vital role in controlling the two-way movement of nutrients and metabolic waste between the choroidal blood flow and the photoreceptors (23). In this role, the RPE serves as an essential guardian of the outer layer of the retina. It consists of a unique monolayer of hexagonally shaped epithelial cells situated between the photoreceptor (PR) cells and Bruch’s membrane (Figure 1) (24). The RPE forms a direct structural and functional interface with adjacent photoreceptors through microvilli. Its distinctive dark brown pigmentation is attributed to melanin, which provides photoprotection to retinal cells by absorbing light and exhibiting antioxidant activity (25). RPE cells possess phagocytic capabilities, allowing them to engulf and clear degenerated photoreceptor outer segments. This process is essential for maintaining retinal homeostasis and ensuring the efficient turnover of cellular components, thereby supporting optimal visual function (26). Additionally, the RPE cells serve as secretory entities in the retina, releasing a range of cytokines and growth factors essential for upholding retinal integrity and maintaining the immune-privileged condition of the eye (27). Moreover, the external basement membrane of the retinal pigment epithelium folds inward and forms structural links with Bruch’s membrane and the choroid (28). Proteins that form tight junctions, essential for the blood-retinal barrier, promote communication between retinal pigment epithelial cells (27). The deterioration of RPE could lead to the loss of rod photoreceptors and subsequent visual impairment (29).

In the retina’s outermost layer (Figure 1), RPE are tightly bound to the Bruch’s membrane beneath them (30). Structurally, Bruch’s membrane consists of five distinct layers (31). The basement membrane is characterized by a delicate and compact extracellular matrix (ECM) network, typically measuring approximately 0.14–0.15 μm in thickness. It primarily comprises laminin, type IV collagen, heparan sulfate proteoglycans, nidogen, and various other glycoproteins. In contrast, the collagenous layer is thicker and has a more open structure, with a thickness that varies from 0.7 to 1.4 μm. This layer is made up of fibrillar collagens, including types I, III, and V, which are integrated with glycosaminoglycans, fibronectin, and proteins from the complement system. Meanwhile, the elastic layer is predominantly composed of several layers of elastin fibers that are arranged in a linear fashion and interconnected (32). It also incorporates type VI collagen, fibronectin, and additional related proteins (33). Owing to its intricate multilayer structure, the Bruch membrane serves as a sophisticated extracellular matrix, which not only supports but also regulates the homeostasis of the outer retina. A key role it plays is facilitating the diffusion and bidirectional transfer of ions, molecules, and nutrients between the RPE and the choroid. Additionally, the Bruch’s membrane demonstrates elastic characteristics and serves a vital mechanical function in resisting intraocular pressure, which may aid in various biomechanical roles linked to ocular adjustments during visual activities (34).

In addition to close connections, RPE cells are also interconnected through adherens junctions and desmosomes, and are connected to the basement membrane/Bruch membrane through focal adhesions (integrin-adhesion complexes) (35). Adherens junctions are mainly connected to the intracellular actin network by cadherin-catenin complexes, while desmosomes rely on desmoglein/desmocollin and intermediate filament connection proteins (such as desmoplakin) to provide stronger mechanical coupling. Focal adhesions interact with the ECM (such as Bruch’s membrane components) through integrins and transfer external forces to the cytoskeleton via molecules such as vinculin, talin, and FAK. These various connection forms together constitute the mechanical conduction pathways between cells and between cells and the matrix, and are key structures for mechanical signal transmission and perception. These mechanical couplings not only affect cell morphology and adhesion stability, but also activate downstream mechanical-sensitive signaling pathways (such as FAK, Rho family GTPases, YAP/TAZ, etc.), and then affect gene expression and epigenetic state by changing nuclear morphology or regulating chromatin conformation.

**Figure 2:**
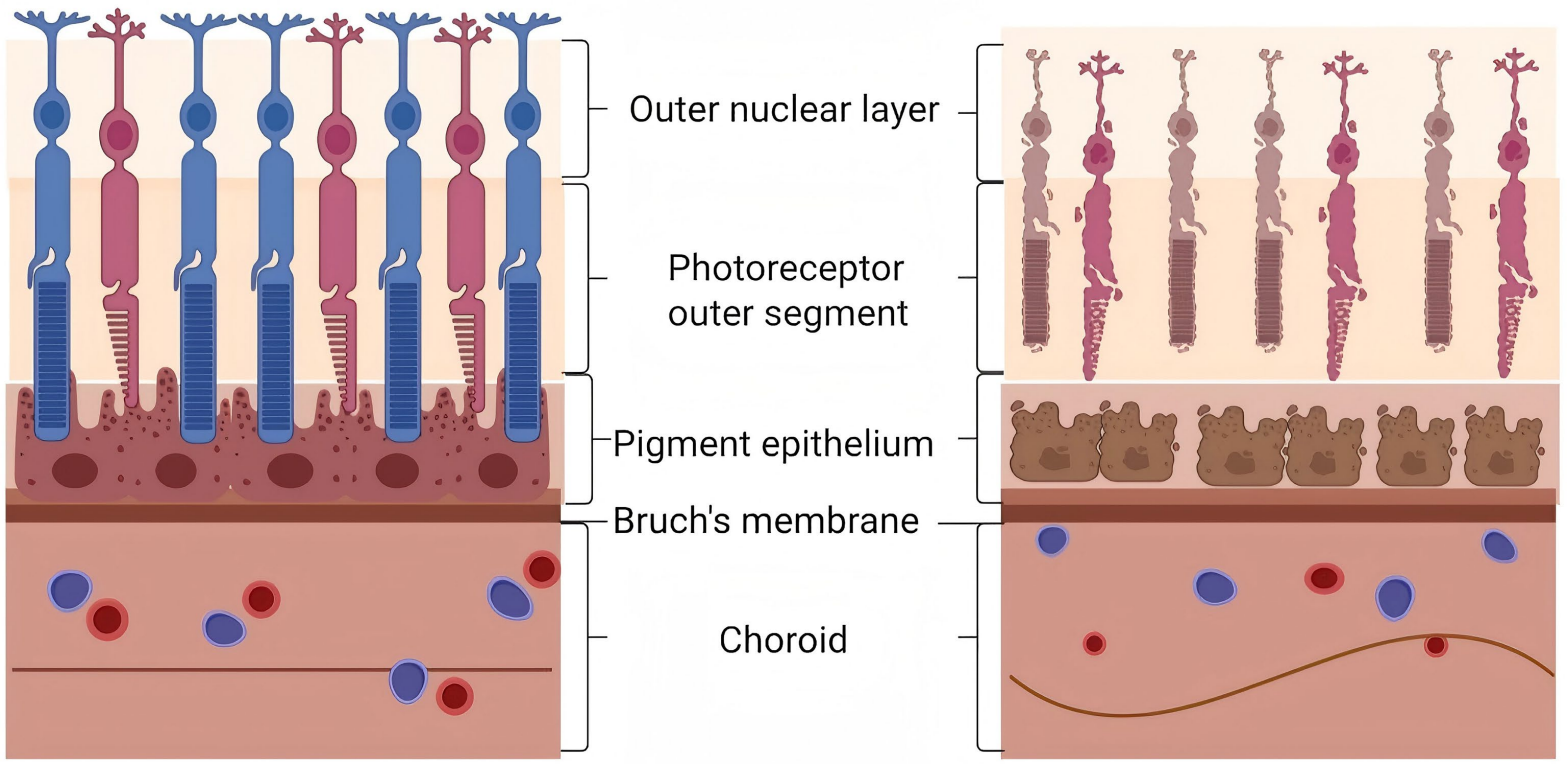
The outer layer of the retina in a pathological state. The figure presents a side-by-side comparison of the outer retinal layers under normal conditions (left panel) and in the context of pathological structural changes (right panel). In the pathological retina (right), several degenerative alterations are evident. Photoreceptor outer segments appear structurally disrupted and shortened, with loss of the orderly disc arrangement. The RPE cells exhibit hypertrophy, irregular morphology, and pigmentary changes consistent with RPE dysfunction. Bruch’s membrane shows visible undulation or thinning, reflecting mechanical stress associated with progressive axial elongation. The choroid demonstrates reduced thickness and attenuated vascularity. Created with BioRender.com.

As retinal degeneration begins, Bruch’s membrane experiences significant restructuring, which results in changes to the adhesion sites between cells and the ECM, potentially affecting the function of the epithelium (33). Due to its acellular nature, the transport of biomolecules across the Bruch’s membrane occurs predominantly through passive mechanisms, with greater molecular exchange observed in the macular region compared to the peripheral areas, which have lower metabolic and functional demands (36). Bruch’s membrane remodeling is primarily characterized by an increase in collagen fiber cross-linking, calcification of elastic fibers, and the turnover of glycosaminoglycans (37). Furthermore, over time, the deeper layers of the Bruch’s membrane gradually gather advanced glycation end products and lipids (38) (Figure 2).

## 3 Mechanical changes of the RPE layer in retinal degeneration caused by high myopia

Cells in the eye encounter various mechanical forces across different types of tissues and anatomical sites (39). The detection and response to mechanical stimuli in these cells occur via mechanosensitive proteins, which transform mechanical signals into biochemical signals within the cell, thereby regulating crucial cellular processes like adhesion, migration, proliferation, and apoptosis (40). As a crucial tissue within the eye, the retina can be subjected to internal or external pressures, such as abnormal traction forces or intraocular pressure. This mechanical force will directly or indirectly affect the function of the retina (41). For example, in retinal detachment (42), abnormal traction forces trigger photoreceptor cell apoptosis via mechanosensitive ion channels, whereas in glaucoma (43), intraocular pressure–induced stress redistributes mechanical strain to the optic nerve head, thereby accelerating the degeneration of retinal ganglion cells. A thorough comprehension of retinal mechanobiology—especially the cellular reactions and signaling pathways influenced by different mechanical stresses during the emergence and advancement of retinal disorders—is crucial for creating innovative therapeutic approaches by manipulating biophysical signals within the retina.

Axial elongation in myopia can excessively stretch the sclera and retina, inducing structural remodeling due to mechanical tension (44). This traction may lead to retinal and macular damage under pathological conditions (45). The RPE is made up of a solitary layer of pigmented epithelial cells, functioning as an essential anatomical barrier that separates the neuroretina from the choroid. The role of the retinal pigment epithelium (RPE) in the phagocytosis of outer segments of photoreceptors, the transportation of vitamin A to the retina, the interchange of metabolic substances, and the management of nutrient transfer between the retina and choroidal circulation has been firmly established (46). The density of retinal pigment epithelial (RPE) cells in the peripheral fundus decreases with increasing axial length of the eye, whereas RPE cell density in the macular region remains largely unaffected by axial elongation (47). This observation is consistent with the hypothesis that axial elongation primarily results from the expansion of the central and peripheral regions of the ocular fundus, particularly Bruch’s membrane. Furthermore, the findings support existing evidence indicating that both the thickness and extent of BM in the macular area are independent of axial length (48). The RPE at the optic nerve head expands, resulting in an increase in the axial length of the eye. This elongation contributes to the formation of the parapapillary *β*-zone in myopia, which is defined as the region of Bruch’s membrane not covered by RPE. This myopic β-zone must be distinguished from the β-zone observed in glaucoma (49). Following the development and expansion of the β zone in myopic eyes, secondary abnormalities may arise in the RPE layer of the macular region, resulting in patchy atrophy—a hallmark feature of stage 3 myopic macular degeneration (50). *In vitro* studies have demonstrated that mechanical stretching can induce alterations in actin filament organization within RPE cells, thereby affecting cytoskeletal dynamics and influencing cellular behaviors such as proliferation and migration (51).

**Figure 3:**
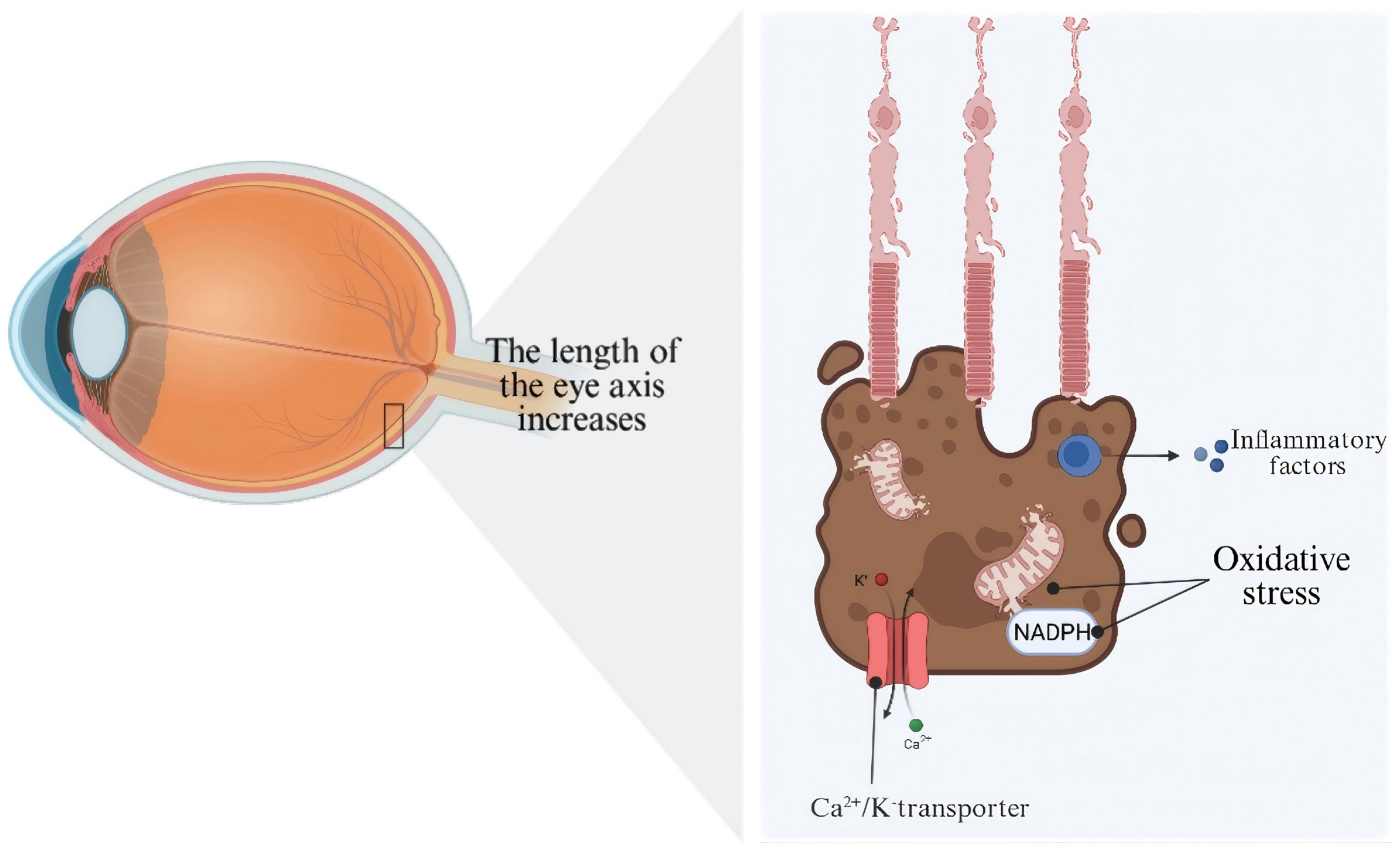
Schematic illustration of retinal pigment epithelium dysfunction in the context of myopia-associated axial elongation. The left panel depicts a cross-sectional overview of a myopic eye, in which pathological increase in axial length — defined as the distance from the anterior corneal surface to the RPE — subjects the posterior ocular tissues to sustained biomechanical stretch. The boxed region demarcates the area of the outer retina shown in magnification in the right panel. The right panel illustrates the cellular consequences of axial elongation-induced mechanical stress on RPE cells (brown), which are shown in close anatomical apposition to the photoreceptor outer segments (pink) above. Under conditions of chronic mechanical strain associated with myopic axial elongation, RPE cells undergo a constellation of pathological changes, including: (1) oxidative stress, characterized by the activation of NADPH oxidase (NADPH) — a key enzyme driving reactive oxygen species (ROS) generation — leading to intracellular oxidative damage; (2) dysregulation of ion transport, as depicted by disruption of the Ca^2+^/K^+^ transporter system at the basolateral membrane, with aberrant flux of potassium ions (K^+^, red circle) and calcium ions (Ca^2+^, green circle) contributing to ionic imbalance and impaired RPE homeostatic function; and (3) inflammatory signaling, whereby activated RPE cells release inflammatory factors (blue circles) into the surrounding microenvironment, potentially exacerbating photoreceptor and choroidal damage. Created with BioRender.com.

In recent times, an increasing amount of studies has shown that the mechanical stretching of RPE cells triggers the expression of genes and proteins linked to cell death, such as Bcl-2 and p53 (52). Liang extended this research and demonstrated that cyclic stretching induces mitochondrial and NADPH oxidase-derived oxidative stress in RPE cells, thereby accelerating oxidative damage (53). Nevertheless, the stimulation of Ca2 + −dependent K + channels was noted in RPE cells subjected to stretching, and this may provide a protective benefit for those cells (54). It is noteworthy that RPE cells exhibit increased secretion of angiogenic proteins and pro-inflammatory cytokines under conditions of sustained mechanical stretching, thereby promoting angiogenesis and potentially contributing to the onset and progression of choroidal neovascularization (55). A study indicates that although periodic stretching leads to pathological alterations, RPE retain the capacity to recover their normal function following the cessation of mechanical strain (52). These findings indicate that mechanical forces may induce pathological alterations through the dysregulation of cytoskeletal signaling pathways in RPE cells at the molecular level (Figure 3).

## 4 The relationship between mechanical memory and epigenetics

Mechanical memory refers to the ability of cells to retain the effects of mechanical stimuli over an extended period following the cessation of such stimuli (56). The cell nucleus and chromatin are capable of retaining a memory of the mechanical response to transient mechanical stress lasting several minutes to hours, and can even propagate this memory to progeny cells following the cessation of stress (57). Experimental findings indicate that the improved diffusion rates of nucleoplasm, chromatin, and RNA polymerase II (RNA Pol II), as well as heightened activity of RNA Pol II and the corresponding gene expression, continue to remain significantly elevated even after stress has ended. These results offer a mechanistic rationale for the prolonged enhancement of transcription seen in cells for several minutes after the cessation of external disturbances. Importantly, the transcriptional activity of RNA Pol II produces a mechanical force around 10 piconewtons, which affects the dynamics of loci and plays a role in the simultaneous presence of fluid-like and solid-like mechanical characteristics within chromatin (58). Research investigating stem cells grown on substrates of differing stiffness has shown that the intracellular retention of substrate mechanical properties is mediated by the mechanosensitive regulators Yes-associated protein (YAP) and TAZ—specifically, Yes-associated protein (YAP) and the transcriptional co-activator with PDZ-binding motif (TAZ)—in conjunction with the pre-osteogenic transcription factor RUNX2 (59). Recent studies employing the nuclear-cytoplasmic transport fluorescence sensor (Sencyt) in monolayer epithelial and mesenchymal cells have demonstrated that YAP functions as a sensor of cell density, yet remains unaffected by nuclear tension, deformation, or structural robustness—mechanical factors known to influence the nucleocytoplasmic shuttling of transcriptional regulatory factors (60). In the study of RPE cells, YAP has been shown to promote the epithelial-mesenchymal transition process and contribute to fibrosis in RPE cells (61). Furthermore, the loss of Yap in RPE cell of adult mice results in the disruption of cellular characteristics (62). The RhoA/YAP signaling pathway is implicated in matrix stiffness-induced fibrosis in the RPE (63).

At both the nuclear and chromatin levels, the preservation of mechanical memory occurs via the structural reorganization of chromatin and changes in the trimethylation of histone H3 at lysine 9 (H3K9me3) (64). For example, cellular responses to extracellular matrix stiffness, compressive forces, and hydrostatic pressure can modulate the activity or expression of chromatin-modifying factors—including histone acetyltransferases, histone deacetylases, and acetyl-CoA—leading to changes in chromatin architecture and gene transcription (64, 65). Linker histone H1.0 is a chromatin-associated protein that mediates the relationship between cellular mechanical properties and chromatin compaction. It regulates gene expression by modulating transcriptional activity and influences the site-specific deposition of histone H3K27Ac, a well-established marker of active genes and enhancers, thereby contributing to the organization of higher-order chromatin architecture within the nucleus (66).

Because the cell membrane and the actin cytoskeleton beneath it are the main targets of mechanical forces generated by the cell and its surrounding environment, mechanical memory initially forms in the actin layer (67). Cells migrating through confined spaces retain a long-term memory of their previously restricted morphology by remodeling the actin cortex and activating the RhoA/ROCK signaling pathway (68). In photoreceptors, the outer segment must initiate cytoplasmic signal transduction involving Rac1 and focal adhesion kinase (FAK)/MERTK, while simultaneously suppressing the activity of RhoA/ROCK (69). This regulation enables F-actin dynamics to facilitate the phagocytosis of outer segment fragments. Subsequently, F-actin-dependent transport mediates the intracellular movement of phagosomes that have been engulfed by RPE cells (69).

Mechanical memory may pose significant challenges for the therapeutic application of *in vitro* propagated cells in tissue regeneration (70). For instance, chondrocytes expanded over multiple generations on two-dimensional rigid substrates retain an epigenetic memory of substrate stiffness, even after transplantation into a three-dimensional *in vivo* environment (64). This retention may diminish their therapeutic efficacy. Recent studies have demonstrated that chromatin and the nucleoplasm can maintain a mechanical memory of protein diffusion rates for several tens of minutes following the cessation of external force (57). This finding supports the notion that mechanical memory may play a critical role in the pathogenesis of various diseases and in modulating treatment outcomes.

It is important to note that most experimental literature on “mechanical memory” comes from studies of acute or short-term mechanical perturbations (with time scales ranging from minutes to hours, or longitudinal observations of several generations of cells). These studies are typically conducted in vitro or in simplified tissue models. In contrast, the mechanical environment in high myopia is usually chronic and progressive (with time scales ranging from several weeks to several years), accompanied by matrix (ECM) remodeling, changes in tissue geometry, chronic inflammation, and cell renewal/selection processes. These factors qualitatively alter the path of force transmission and the establishment of epigenetic homeostasis. Therefore, although acute mechanical memory studies help identify mechanical sensing pathways that may be involved in the retina of myopia (such as integrin/FAK, Rho GTPases, YAP/TAZ, nuclear deformation and chromatin compression, etc.), caution should be exercised when directly extrapolating short-term experimental results to continuous pathological mechanical loads. To determine whether the same mechanism plays the same role in short-term memory and long-term, disease-related epigenetic reprogramming, longitudinal in vivo models and long-term mechanical stimulation experimental paradigms are still needed for verification.

Mechanical memory, as used here, refers to a persistent cellular state in which prior mechanical cues (e.g., substrate stiffness or externally applied force) induce molecular/epigenetic alterations that remain detectable after the mechanical stimulus is removed, on timescales ranging from minutes to days depending on the model. This phenomenon may challenge the therapeutic use of in vitro–expanded cells for tissue regeneration (68). For example, chondrocytes expanded over multiple passages on rigid two-dimensional substrates have been reported to retain stiffness-associated epigenetic features even after transplantation into a three-dimensional in vivo environment (62), which may compromise their functional performance. In addition, studies in chromatin/nucleoplasm mechanics have shown that certain force-induced changes—such as altered protein diffusion behavior—can persist for tens of minutes after cessation of external force (55), consistent with a short-term form of mechanical memory at the nuclear level. Importantly, these observations are derived primarily from non-ocular systems (e.g., cartilage/mesenchymal or nuclear biophysics models), and direct evidence demonstrating mechanical memory in retinal or RPE tissue is currently limited. Therefore, the relevance of mechanical memory to retinal/RPE biology or myopia-related pathology should be considered a testable hypothesis, and future work will need to define tissue-specific time windows and molecular markers in ocular models and human samples.

## 5 Epigenetic regulation of the outer retina in high myopia

Although a growing number of epigenetic alterations have been documented in the outer retina during high myopia (for example changes in DNA methylation, specific histone marks, chromatin accessibility and RNA modifications), direct evidence that these alterations are driven by mechanical forces in myopic eyes is currently limited. To guide mechanistic interpretation and future experiments, we therefore summarize plausible pathways by which mechanical cues could induce the observed epigenetic changes. Mechanotransductive inputs at the cell surface and cytoskeleton (integrin/FAK complexes, focal adhesions, actomyosin contractility and RhoA signaling) can modulate nuclear shape and tension and thereby influence chromatin compaction and the activity/localization of chromatin modifiers (e.g., histone acetyltransferases, deacetylases, methyltransferases). YAP/TAZ signaling—well established as responsive to ECM stiffness and cell geometry—can act as a transcriptional co-activator and recruit chromatin remodelers to specific loci, potentially linking persistent mechanical loading to stable changes in histone marks and gene expression. Additionally, mechanical stress can alter nucleocytoplasmic transport and the mobility of transcriptional machinery, which may affect co-transcriptional processes including RNA modifications (such as m6A). We emphasize that these links are largely inferred from studies in other cell types and tissues; where retinal/RPE-specific data are lacking we flag the statements as hypotheses and propose experimental approaches to validate them in the context of high myopia (see below).

### 5.1 Non-coding RNA

#### 5.1.1 micRNA

Clinical studies have demonstrated that the expression level of has-miR-328-3p in the blood of patients with myopic macular degeneration is elevated, which is accompanied by a reduction in the optical density of the RPE layer (71). The C allele of rs662702 in the PAX6 gene is associated with an increased risk of myopia, and the expression of miR-328 in the blood cells of myopic patients is significantly higher compared to that in the control group. Retinoic acid (RA) upregulates miRNA-328 expression and downregulates PAX6 expression (72). Chen et al. further demonstrated that the C risk allele of the 3’UTR SNP rs644242 exhibits a strong interaction with miR-328, leading to the downregulation of PAX6 expression. This downregulation of PAX6 at the transcriptional level promotes increased proliferation of RPE cells, while simultaneously reducing the proliferation of human scleral fibroblasts (HSF) (73). According to Liang, miR-328-3p downregulates protein expression in a dose-dependent manner at the mRNA level. Fibromodulin (FMOD) enhances the phosphorylation levels of p38-MAPK and MAPK8, promoting the expression of TGFB1. Additionally, anti-miR-328-3p suppresses the elongation of AL (74). Myopic patients exhibit upregulation of miR-328 in both peripheral blood and retinal tissues, suggesting that miR-328 may serve as a potential biomarker for myopia.

Recent physical research has further substantiated the findings of Mei et al., who identified significantly elevated levels of miR-466c-5p, miR-466 h-5p, miR-466j, miR-468, miR-669e, miR-15a, miR-16-1, and miR-294 in retinal and whole-eye samples from form-deprivation myopia (FDM) animal models (75). Among these, seven miRNAs were notably enriched in biological processes associated with transcriptional regulation, axon guidance, and the TGFB1 signaling pathway. Furthermore, a subsequent study indicated that the expression levels of seven upregulated miRNAs—miR-101a, miR-6690, miR-466f, miR-291a, miR-465b, miR-696, and miR-18b—in the retinas of FDM mice were fivefold higher than those in the sclera, suggesting their potential involvement in retina-specific regulatory mechanisms (76). Importantly, although miR-145 was found to be downregulated in a subset of retinal cells, its overall expression in the retina was observed to be 25.4 times higher than that in the sclera, indicating a potentially significant functional role in retinal physiology (76).

Liu demonstrated that the transcription of miR-92b-3p is down-regulated in guinea pigs with lens-induced myopia (LIM), which leads to an increased expression of p53 and the BTG antiproliferative factor 2 (BTG2). The upregulation of BTG2 induces apoptosis in retinal tissue cells, promotes DNA damage within the retinal tissue of guinea pigs, enhances the expression of BCL2 apoptosis regulator (BCL2) and BCL2-associated X protein (BAX), and elevates levels of cyclin-dependent kinase 2 (CDK2). These molecular alterations contribute to a reduction in retinal thickness, electrophysiological dysfunction, and impaired visual function (77).

The expression levels of miR-182-5p, miR-181a-5p, miR-183-5p, miR-9-5p, and miR-96-5p are elevated in the LIM mouse model. Overexpression of miR-181a-5p has been implicated in the development of myopia and myopic retinopathy. Furthermore, miR-181a-5p can induce autophagy, promote the proliferation of RPE cells, and directly target N-sulfoglucosamine sulfohydrolase (SGSH) in ARPE-19 cells (78). A highly conserved miR-183/−182/−96 miRNA cluster is expressed as a single polycistronic transcript and exhibits significant sequence homology in its target sites, thereby regulating a microphthalmia-associated transcription factor that is essential for the differentiation of RPE cells in mice (79).

In both FDM and LIM animal models of the retina, Cui et al. demonstrated that mutations in the TEA domain family member 1 (TEAD1) may lead to the down-regulation of miR-671-5p expression. This down-regulation potentially impacts the hub genes cyclic adenosine monophosphate response element binding protein 1 (CREB1) and MAPK1, which are critically involved in extracellular estrogen signaling and visual learning pathways. Furthermore, atropine has been shown to target MAPK1 and CREB1, which are key components of the retinochoroidal signaling cascade (80). Additionally, Liu reported that the expression levels of mmu-miR-1936, mmu-miR-673-3p, and mmu-miR-338-5p were significantly upregulated in the retinas of mice with FDM. The target genes of these microRNAs, including Pax6 and SMAD family member 3 (SMAD3), are predominantly involved in biological processes such as retinal tissue morphogenesis and developmental growth. By regulating the expression of genes through transcription, these three microRNAs may contribute to the onset process of myopia in mice (81).

A study has confirmed the impact of alterations in DNA methylation and microRNA (miRNA) expression on myopia in children (82). Research in Young Children has identified the promoter regions of MIR3621, MIR34C, and MIR423—genes exhibiting elevated methylation levels—and MIR1178, MIRLET7A2, MIR885, MIR548I3, MIR6854, MIR675, MIRLET7C, and MIR99A—genes showing decreased methylation levels—as differentially methylated at CG dinucleotides. Several targets of these miRNAs, including GNAS, TRAM1, CTNNB1, EIF4B, TENM3, and RUNX, have been previously associated with high myopia (HM) and refractive errors in European populations through genome-wide association studies. Overrepresentation analysis of miRNA target genes has revealed significant enrichment in biological pathways and processes related to eye structure and function, such as axon guidance, transcription regulation, cell adhesion, and signaling pathways involving TGF-*β*, insulin, MAPK, and EGF-EGFR. Follow-up investigations by the research team suggest that alterations in specific CG dinucleotide methylation patterns may be linked to early-onset high myopia (83).

#### 5.1.2 lncRNA

Research on myopia-related long non-coding RNAs (lncRNAs) primarily involves the sequencing of ocular tissues from animal models to identify target genes, enriched functional networks, and specifically expressed lncRNAs. Li identified 655 lncRNAs that exhibit distinct expression patterns in the retinas of FDM models (84). These lncRNAs are primarily enriched in biological processes related to retinol metabolism, cytokine-cytokine receptor interactions, cellular activities, and rhythmic regulatory pathways. Notably, the lncRNA Gm35369 is predominantly expressed in horizontal cells in mice (84).

Compared to the healthy control group, Geng et al. observed aberrant expression of posterior pole long non-coding RNA (lncRNA) in the eyes of guinea pigs with FDM and LIM. This dysregulated expression was primarily associated with cellular components such as ECM structural constituents, molecular functions including kinase activity, metabolic processes, and regulation of cell growth, as well as biological pathways (85). Based on the concept of molecular structure effects proposed by Wang et al., lncRNAs adopt complex three-dimensional conformations that serve as scaffolds for recruiting transcription factors (TFs). Single nucleotide variants affecting critical functional domains and alternative splicing regions were identified within the transcription factor binding sites (TFBSs) of myopia-associated lncRNA transcripts. These genetic alterations induce substantial conformational changes in the lncRNA structure, thereby modulating the onset and progression of myopia in human (86).

#### 5.1.3 circRNA

Circular RNA (circRNA) has been shown to play a significant role in the pathogenesis of various ocular diseases (87). In the scleral tissue of mouse models with FDM, altered circRNA expression profiles have been observed. Specifically, the regulatory axes circPank1/miR-145-5p/NRAS and circNbea/miR-204-5p/ITPR1 have been identified, suggesting their potential involvement in the progression of myopia (88). In vitreous humor samples from individuals with high myopia, Pearson correlation analysis and multivariate regression analysis revealed a positive and significant association between ocular axial length and has-circNbea as well as has-circPank1 (88). KEGG pathway enrichment analysis indicated that the target genes of circRNAs were predominantly involved in the mTOR, insulin, cAMP, and VEGF signaling pathways. GO functional enrichment analysis suggested that these circRNAs primarily regulate biological processes related to transcription, cellular components such as the cytoplasm, and molecular functions including protein binding. Furthermore, circRNA-mediated ceRNA network construction and PPI network analysis identified several hub genes potentially implicated in the pathogenesis of myopia (88) (Table 1).

**Table 1:** Key studies of non-coding RNAs and epigenetic regulation in myopia.

Author	Model	Mechanical stimulus	Epigenetic readout	Main finding	Limitations
Chen et al. (2012) (73)	Human (peripheral blood cells), ARPE-19 (cell line)	*N/A* (Genetic association; retinal stretch implied via axial elongation in patients)	miR-328-3p ↑; SNP rs662702 (PAX6 3′UTR)	C allele of rs662702 disrupts miR-328 binding → ↓ PAX6 expression → ↑ RPE proliferation & ↓ scleral fibroblast proliferation	Clinical correlation only; no direct mechanical perturbation tested; cell line may not reflect in vivo RPE function
Liang et al. (2022) (74)	Mouse (FDM model); ARPE-19 cells	FDM; *in vitro* RA treatment as surrogate for metabolic stress	miR-328-3p ↑ (dose-dependent); FMOD ↓; p38-MAPK/MAPK8 ↑; TGFB1 ↑	Anti-miR-328-3p suppresses axial elongation (AL); miR-328-3p targets FMOD → activates TGF-β1 pathway	RA not a direct mechanical stimulus; anti-miR efficacy tested only in mice, not primates/humans;
Mei et al. (2017) (75)	Mouse (FDM)	FDM; (goggle-induced)	8 miRNAs ↑: miR-466c-5p, −466 h-5p, −466j, −468, −669e, −15a, −16-1, −294	Enriched in transcriptional regulation, axon guidance, TGF-*β* signaling	No functional validation; tissue-level RNA-seq without cell-type resolution
Tkatchenko. (2016) (76)	Mouse (FDM)	FDM	7 miRNAs ↑ in retina vs. sclera (5×): miR-101a, −6,690, −466f, −291a, −465b, −696, −18b; miR-145 ↓ in subset but 25.4 × higher in retina vs. sclera	Retina-specific miRNA enrichment suggests compartmentalized regulatory roles in myopia	miRNA quantification not normalized to housekeeping genes; functional assays lacking
Liu et al. (2024) (77)	Guinea pig (LIM: −10 D lens)	LIM	miR-92b-3p ↓	Downregulation → ↑ p53 & BTG2 → apoptosis, DNA damage, ↓ retinal thickness & visual function	Single miRNA focus; no rescue experiments with mimics/inhibitors
Jiang et al. (2024) (78)	Mouse (LIM); ARPE-19 cells	LIM	miR-181a-5p ↑; miR-182/183/96 cluster ↑	miR-181a-5p → ↑ RPE proliferation & autophagy; targets SGSH; cluster regulates MITF → RPE differentiation	ARPE-19 is immortalized; lacks native RPE polarity/microenvironment
Cui et al. (2023) (80)	Mouse (FDM & LIM)	FDM& LIM	miR-671-5p ↓ (TEAD1 mutation-linked)	↓ miR-671-5p → dysregulation of CREB1/MAPK1 (estrogen & visual learning pathways); atropine targets these hubs	TEAD1 mutation not naturally occurring in myopia; overexpression/knockout not performed
Liu et al. (2022) (81)	Mouse (FDM)	FDM	mmu-miR-1936, −673-3p, −338-5p ↑	Targets Pax6 & SMAD3 → implicated in retinal morphogenesis & growth	Target validation limited to bioinformatics; no luciferase/reporter assays shown
Geng et al. (2020) (85)	Guinea pig (FDM & LIM)	FDM& LIM	Posterior pole lncRNAs dysregulated	Associated with ECM structure, kinase activity, cell growth regulation	Lack of lncRNA knockdown/rescue; unclear if changes are cause or consequence
Wang et al. (2021) (86)	Human (bioinformatic + in silico)	*N/A* (Computational modeling)	SNVs in TFBS of myopia-associated lncRNAs	SNVs induce lncRNA 3D structural changes → alter TF recruitment → modulate myopia	Purely computational; no experimental validation in cells/animals
Zhang et al. (2024) (88)	Mouse (FDM sclera); Human (vitreous humor, HM)	FDM (sclera); clinical axial elongation (human)	circPank1 ↑, circNbea ↑; miR-145-5p ↓, miR-204-5p ↓	circPank1/miR-145-5p/NRAS & circNbea/miR-204-5p/ITPR1 axes → mTOR/VEGF/cAMP signaling; vitreous circRNAs correlate with AL	Human vitreous data correlative only; no perturbation of circRNAs to test causality.

### 5.2 DNA and RNA methylation

Mechanical regulation could modulate DNA methylation patterns indirectly via mechanosensitive control of DNA methyltransferases or through long-term changes in cell state (for example fibrosis or altered cellular composition) that select for cells with distinct methylomes. Testing whether sustained mechanical strain alters DNMT expression/activity in RPE/retina would clarify this possibility.

DNA methylation generally leads to gene silencing, whereas DNA demethylation is associated with increased gene expression. The precise balance between continuous methylation and demethylation activities determines the cell’s final methylation pattern (89). Moreover, a study conducted on Chinese adolescents revealed that the methylation level of the PAX6 gene was slightly elevated in individuals with myopia, suggesting that PAX6 gene methylation may serve as a highly accurate predictor for mild myopia (90). m^6^A modification represents a major epigenetic regulatory mechanism and has been implicated in the degenerative processes of the retina associated with high myopia (89). Clinical evidence indicates that blood m^6^A levels are significantly inversely correlated with the severity of myopia (91).

Wen (92) conducted methylated RNA immunoprecipitation (MeRIP) sequencing, revealing that the m^6^A methylation level in the anterior capsule transcriptome of individuals with high myopic cataract was significantly elevated compared to non-myopic cataract patients. Furthermore, the m^6^A modification level of chitinase 3-like protein 1 (CHI3L1) mRNA was markedly increased in patients with high myopic cataract relative to non-myopic controls, while the level of fibroblast growth factor 10 (FGF10) mRNA was decreased (92). Additionally, integrated analysis of MeRIP and mRNA sequencing indicated that the expression of the methyltransferase METTL14 was upregulated, whereas the expressions of the demethylases FTO and ALKBH5 were downregulated. These molecular alterations may collectively contribute to the hypermethylation of the CHI3L1 transcript, potentially enhancing the abundance of the encoded protein and promoting extracellular matrix remodeling (92).

FGF10 (93) regulates RNA metabolism through its interaction with angiogenesis-related RNA-binding proteins, particularly RNA binding motif protein 10 (RBM10). In patients with high myopia and cataract, hypomethylation of FGF10 transcripts may lead to reduced FGF10 protein abundance and a diminished interaction between FGF10 and RBM10, thereby increasing the risk of choroidal neovascularization in individuals with high myopia. A study conducted by Swierkowska on children with high myopia identified significant hypomethylation at the cytosine-phosphate-guanine (CpG) site within the CpG island at the 5q31 locus, which is located in the promoter region of the protocadherin α10 (PCDHA10) gene cluster. This region overlaps with the intron of PCDHA19 (83). Such epigenetic alterations may contribute to the early onset of high myopia (83). Furthermore, experimental models of myopia have demonstrated that genes associated with myopia progression can upregulate their expression through the hypomethylation of promoter regions. In a guinea pig model of FDM, Ding observed that hypomethylation at four CpG sites within the promoter region of the insulin-like growth factor 1 gene was associated with increased IGF-1 transcriptional activity in the sclera, contributing to the development of myopia (94).

Based on these findings, further examination of changes in the outer retinal layer revealed that a research team in Singapore observed reduced methylation levels in the promoter region upstream of the homeobox A9 (HOXA9) gene within the RPE in children with myopia (95). This hypomethylation phenomenon is associated with the increased expression of the HOXA9 gene, and the expression of the HOXA9 gene is positively correlated with the expressions of insulin-like growth factor 1 receptor (IGF1R), matrix metalloproteinase 2, fibroblast growth factor 2, and transforming growth factor *β* (95). These molecular factors contribute to RPE proliferation, axial elongation of the eye, and the progression of myopia (95).

### 5.3 Histone acetylation

Forces transmitted to the nucleus can change chromatin compaction and accessibility and influence the recruitment or activity of histone modifiers (e.g., HATs, HDACs, HMTs). Persistent mechanical loading might therefore stabilize histone-mark changes (for example H3K9me3, H3K27ac) observed in high myopia. Experiments combining controlled mechanical perturbation with ChIP-seq/ATAC-seq in RPE/retinal cells could directly test this link.

Acetylation is a common and reversible post-translational modification of proteins, enzymatically regulated by two opposing classes of enzymes: lysine acetyltransferases (KATs), which catalyze the transfer of acetyl groups to lysine residues, and lysine deacetylases (KDACs), which remove these acetyl groups (96).

Studies have shown that the glycolytic activity in the optic nerve sheath region of mouse embryos is significantly enhanced. During the developmental stage of retinal progenitor cells, if the lactate dehydrogenase A and solute carrier family 2 member 1 genes are knocked out through genetic intervention, it will lead to the obstruction of eye development (97). Furthermore, histone H3 acetylation—a key epigenetic modification—is regulated by histone deacetylase (HDAC) in a lactate-dependent manner, thereby modulating the expression of critical region-specific transcription factors (97). According to the study by Park et al., the expression levels of HDAC2 and HDAC3 in the extracellular matrix of the limbal conjunctiva (LC) and peripheral sclera (PPS) among South Koreans are lower compared to those observed in Caucasians. In contrast, there are differences in the acetylation status of histone components in elastic fibers and the promoter region of lysyl oxidase-like protein 2 (LOXL2) among different races. Among them, the expression levels of acetylated histone H3, elastic fiber components, and LOXL2 in Koreans are higher than those in Caucasians (98). A study conducted on Chinese people suggests, HM is associated with the voltage-gated potassium channel subunit KCNQ5, which is encoded by a gene located in a genomic region encompassing or adjacent to DNase I hypersensitive sites and histone modification markers H3K27ac and H3K4me1. Single nucleotide polymorphisms rs7744813 and rs9342979 are situated within this regulatory region, suggesting a potential role in gene expression modulation. These findings indicate that KCNQ5 may serve as a genetic risk factor for HM, with its polymorphisms contributing to the heritable susceptibility to the condition. Furthermore, histone acetylation, particularly at H3K27ac, has been implicated in ocular development and the pathogenesis of HM (99).

Histone modifications present promising avenues for the development of novel therapeutic targets in the treatment of myopia. Their reversible nature allows these modifications to serve as effective tools for regulating gene expression in ocular tissues, thereby enhancing our understanding of the pathophysiology and genetic underpinnings of eye diseases (100). Furthermore, racial variations in histone modification patterns highlight the potential for population-specific targeted therapies. However, the involvement of multiple enzymes and intricate regulatory pathways in histone modifications renders the underlying mechanisms highly complex. Achieving precise control over these processes necessitates substantial research efforts and time. Additionally, the considerable variability in histone modification effects across different racial and individual groups poses a significant challenge in establishing standardized treatment approaches.

## 6 Discussion

Recent advances in epigenetic research have illuminated a potential interplay between genetic predisposition and environmental influences in myopia development (99). Epigenetic mechanisms — including DNA methylation, histone modifications, non-coding RNA regulation, and RNA methylation — have emerged as modulators of gene expression patterns governing refractive development and ocular growth, though current evidence derives predominantly from *in vitro* cell culture systems, ex vivo tissue preparations, and *in vivo* animal models. Although myopia risk is not determined solely by genetic variants, environmental exposures may shape ocular growth trajectories through epigenetic regulation; whether such alterations are causally involved or stably maintained in human myopia, however, remains to be rigorously established (101).

Altered DNA methylation at loci associated with ocular development and signaling has been linked to myopia-related phenotypes in experimental systems and limited human observational datasets, yet direct corroboration in human ocular tissues — including scleral, retinal, or choroidal biopsies — remains notably scarce. Histone modifications may similarly modulate chromatin accessibility and thereby influence transcriptional programs relevant to eye growth, as indicated by preclinical evidence. Furthermore, ncRNAs — particularly microRNAs — have been identified as regulators of gene expression in cell- and animal-based myopia models, pointing to their potential as molecular targets for attenuating pathological axial elongation.

Mechanical stimuli, which are central to several myopia paradigms (e.g., stretch- and strain-based experimental systems), can drive transcriptional changes alongside chromatin-level regulatory events in vitro and in animal models, potentially culminating in lasting epigenetic remodeling. Nonetheless, these observations should not be extrapolated as established mechanistic principles in human high myopia in the absence of direct human tissue evidence. Future investigations should delineate how mechanical forces initiate epigenetic reprogramming and assess the translational relevance of such changes in human clinical samples and ocular biopsy material, which will be indispensable for advancing therapeutic development (102).

Epigenetic interventions — including demethylating agents and microRNA-based strategies — have demonstrated proof-of-concept efficacy in preclinical settings; however, their safety profiles and therapeutic applicability in humans warrant systematic evaluation. Ultimately, integrative analyses combining genomic, transcriptomic, and epigenomic data — ideally anchored in well-phenotyped human cohorts and disease-relevant ocular tissue datasets — hold promise for deepening our mechanistic understanding of myopia and informing the development of targeted prevention and treatment strategies.

## 7 Limitations and future prospects

Despite the conceptual framework proposed in this review, several important limitations of the current literature warrant explicit acknowledgment. First, the majority of mechanistic evidence cited herein derives from in vitro cell culture systems and non-ocular tissues, with direct experimental validation in human retinal or RPE tissue remaining scarce. The extent to which findings from mechanical stretch models, mesenchymal cell systems, or non-mammalian organisms faithfully recapitulate the mechanobiological environment of the human outer retina is uncertain, and cross-tissue or cross-species inferences should therefore be interpreted with appropriate caution. Second, direct longitudinal human data linking progressive mechanical changes in the myopic eye — such as increasing axial length, Bruch’s membrane thinning, or choroidal thinning — to specific epigenetic reprogramming events in defined retinal cell populations are currently absent. Without such data, the proposed mechanistic cascade from ocular biomechanical stress to epigenetic dysregulation and photoreceptor/RPE dysfunction remains largely inferential. Third, a substantial translational gap exists between the animal models most commonly used to study myopia (e.g., form-deprivation or lens-defocus paradigms in mice, guinea pigs, or chicks) and human high myopia, particularly with respect to species-specific differences in scleral composition, retinal organization, and the biomechanical consequences of axial elongation; findings from these models may not directly translate to the human disease context. Fourth, establishing causality between mechanical stimuli and epigenetic outcomes *in vivo* remains methodologically challenging. Most available evidence is correlative in nature, and disentangling the contributions of mechanical stress from concurrent metabolic, inflammatory, oxidative, or genetic factors in the myopic retinal microenvironment represents a significant unresolved challenge. Collectively, these limitations underscore that the mechanobiological-epigenetic axis in high myopia, while conceptually compelling and supported by convergent indirect evidence, should currently be regarded as a working hypothesis requiring direct experimental validation in ocular-specific and human-relevant models.

A key limitation of the current literature is the absence of direct, longitudinal evidence linking chronic mechanical changes in the myopic eye to specific epigenetic reprogramming events in retinal cell types. To address this, we propose four experimental directions: (1) long-term mechanical loading models (days to weeks of stretch/compression or variable substrate stiffness applied to RPE/outer nuclear layer cells) with longitudinal tracking of DNA methylation, histone modification, chromatin accessibility, and RNA modification; (2) time-series sampling of induced myopia animal models, combining epigenomic and transcriptomic analyses to capture epigenetic changes concurrent with axial elongation; (3) causal intervention studies using gain- and loss-of-function approaches targeting mechanosensing molecules (integrins/FAK, RhoA/ROCK, YAP/TAZ) or epigenetic enzymes (DNMTs, HMTs, HATs, m^6^A writers/erasers) to determine whether epigenetic changes can be blocked or recapitulated independently of mechanical input; and (4) single-cell and spatial omics approaches to resolve mechanically induced epigenetic alterations at the level of specific cell subpopulations and anatomical locations, minimizing confounding from tissue heterogeneity.
